# Hazard Identification and Risk Assessment Using Energy Tracing and Barrier Analysis Method in the Imaging Department of Amir al-Momenin Ali (AS) Hospital in Gerash City -

**DOI:** 10.31661/gmj.v14i.3904

**Published:** 2025-12-03

**Authors:** Amir Bahardoost, Sanaz Khoramipour, Behnoosh Khoshmanesh

**Affiliations:** ^1^ Department of Environment, Wt.c., Islamic Azad Univercity, Tehran, Iran

**Keywords:** Hazard Identification, ETBA Method, Risk Assessment, Hospital

## Abstract

**Background:**

Hospitals are considered one of the most important institutions providing health services; given the presence of specific hazards, compliance with safety principles is of utmost importance. This study aimed to identify and evaluate the risk of existing hazards using the Energy Tracing and Barrier Analysis (ETBA) method in the imaging department.

**Materials and Methods:**

The present study is an analytical study performed to assess the risk in the imaging department of Amir al-Momenin Ali (AS) State Hospital in Gerash in 2024. In this study, potential risks were identified using the ETBA technique and qualitatively assessed via the risk matrix (MIL-STD882E). Observation, interviews with department experts, review of documents, work instructions, technical documents of devices, layout of devices, as well as documents of the maintenance and repair unit of this department were used for data collection.

**Results:**

A total of 433 risks were identified, 232 of which were human-related, 167 were equipment-related and 34 were environmental-related. At the primary risk level, 13 were identified as unacceptable risks (high level) requiring immediate intervention, 281 as undesirable risks (serious level), 79 as acceptable risks with revision (medium level), and 61 as acceptable risks (low level). At the secondary risk level, once the controls were implemented, the results improved and the number of undesirable risks dropped to 18, 274 as medium level, and 141 as low level, demonstrating the effectiveness of the controls in mitigating risks.

**Conclusion:**

In this study, in addition to obtaining the level of risks in the hospital imaging department, the level of possible risks in each of those departments was also measured. Further, based on the existing assessment levels, control measures and suggestions were presented. They included implementing measures such as safety training, periodic inspection monitoring system, operator training, protection control, cable inspection, installation of overload alarms, improving preventive maintenance, as well as providing appropriate educational solutions to control the identified risks.

## Introduction

Accidents are undesired events that cause damage to assets and the organization as a whole. An accident highlights a flaw in the system, after which the defect and ways to establish an optimal situation should be evaluated. Every organization needs an up-to-date and suitable system which is a balanced combination of management, engineering and training methods to control risks as well as accidents [1][2][3]. Hospitals are one of the most important institutions providing health and medical services, and given the presence of specific risks, compliance with safety principles is of great importance. Compliance with safety principles and regulations is performed to prevent or reduce accidents by eliminating and controlling hazards [4].

Safety standards in hospitals can be examined in several domains, including: 1- patient safety, 2- patient and staff safety, 3- equipment safety, 4- physical resource and facility safety [5].

The existence of various hazards in hospitals has been repeatedly mentioned in numerous studies. For instance, hazards include the following: electric shock resulting from the increased use of diagnostic and therapeutic equipment such as electrocardiograms and electrosuction devices, chemical hazards observed after exposure to disinfectants, cleaning compounds, drugs, mercury and anesthetic gases, fire and explosion resulting from increased fire hazards with the development of vertical buildings, as well as the use of pressurized and heated devices, slips and falls owing to unsafe surfaces, radiation exposure following the use of radioactive and radioactive materials for diagnostic and therapeutic purposes, hospital waste produced by microorganisms, needle and sharps injuries along with contamination by pathogens including hepatitis B, C and human immunodeficiency viruses, respiratory disorders and impaired lung function due to exposure to chemicals and bioaerosols, musculoskeletal disorders as one of the most common causes of absenteeism and injury among HCWs, especially women, as well as psychological hazards such as job stress, shift work, and workplace violence [6][7].

Based on results obtained globally, 2.9 million deaths were attributed to work, of which 2.58 million were due to work-related diseases and 0.32 million were because of occupational injuries. Work-related disability-adjusted life years were estimated at 180 million in 2019, which was linked to an economic loss of 5.8% of global GDP [8]. In this regard, a major hazard is radioactive materials and ionizing radiation in hospital units used for diagnosis and treatment of patients. It is located in the imaging department, helping the patient recover by providing imaging services. Indeed, it is regarded one of the diagnostic complexes where a part of the fixed assets and human resources of the hospital are concentrated [9][10].

The ETBA method is based on the logic that the damage caused by an accident occurs due to the unwanted exchanges that take place during the energy flow from the barrier to the exposed targets. Tracing energy and barrier performance is a qualitative analysis used to establish more precise risks. In this method, risks are discovered using the principle of tracing the energy flow in systems or operations. This method is one of the most useful and informative tools available to researchers to evaluate the safety of systems. In this technique, the incident is defined as an unwanted release of energy that occurs in response to inadequate barriers [11][12].

Thus, since Gerash Hospital has an imaging department and such a study has not been performed in this department so far, there has been a need of addressing the safety of medical staff in this department. Accordingly, this study was conducted for identifying hazards and assessing risk using the ETBA method in the imaging department of Amir al-Momenin Ali (AS) Educational and Therapeutic Hospital in Gerash County, with the main focus on evaluating safety risks in the department.

## Materials and Methods

### Study Design and Setting

The present study was a qualitative analytical study conducted to assess risk in the imaging department of Amir al-Momenin Ali (AS) State Hospital in Gerash County in 2024. The study was designed in seven stages: (1) identifying the types of energies present in the system, (2) determining the source of energy generation, (3) tracing energy flow paths, (4) identifying and evaluating existing protections and barriers, (5) identifying vulnerable targets, (6) calculating and classifying existing risks, and (7) proposing risk control measures. In the first step, significant expertise was required to identify various energies, leading to the formation of an Energy Trace and Barrier Analysis (ETBA) team. The team included the head of the imaging department, a radiology expert, an operator, a medical equipment manager, an occupational health engineer, and a researcher.

Data were collected using an energy worksheet and checklist, where the influence of each energy was evaluated separately on potential targets (humans, equipment, and the environment). Primary and secondary risk levels were assessed for each energy. Data collection methods included observations, interviews with department experts, and reviews of documents, work instructions, technical device manuals, device layouts, and maintenance records. An energy identification checklist was used to detect 15 distinct energies, and their flow paths were mapped to identify potential targets and assess their impact. Existing controls—including physical, spatial, temporal, and process controls—were identified, and primary risk levels were calculated. If the primary risk exceeded the hospital’s acceptable threshold, control barriers were proposed, and secondary risk was calculated. Risks were classified using the Risk Assessment Code (RAC) based on the MIL-STD882E standard, categorizing them as unacceptable (high), undesirable (serious), acceptable with revision (average), or acceptable (low). The RAC was determined by combining risk severity and probability to prioritize risks and guide control measure development.

The qualitative risk assessment matrix from MIL-STD882E was used, classifying risk severity into four categories (catastrophic, critical, borderline, minor) and probability into five (A, B, C, D, E). Combining these factors produced a risk matrix to estimate acceptable and unacceptable risk levels. Proposed controls were developed in consultation with facility experts to improve safety and reduce risk.

### Samples

The statistical population included all personnel in the imaging department of Amir al-Momenin (AS) Hospital in Gerash.

This encompassed radiology experts, imaging technicians, radiologists, and other department personnel, as well as imaging equipment such as ultrasound and mammography machines.

The sample consisted of 10 purposively selected experts from the imaging department, ensuring representation of the statistical population. Participants included the head of the imaging department, two radiologists, three radiology experts, one nurse, one occupational health engineer, one receptionist, and one service staff member. These individuals possessed sufficient expertise to contribute effectively to hazard identification and risk assessment using the ETBA method.

### Outcome Measurements

The study utilized several tools for data collection and analysis, including the ETBA energy checklist, risk severity classification (MIL-STD882E), risk probability classification (MIL-STD882E), risk matrix (MIL-STD882E), and risk classification criteria (MIL-STD882E).

The primary outcomes involved identifying energy sources, assessing their risks, and evaluating existing controls. Secondary outcomes included proposing mitigation strategies for unacceptable risks. Data were analyzed based on the checklist responses, with qualitative variables described using frequencies and percentages.

### Statistical Analyses

Data analysis was conducted using the energy checklist and supplementary information obtained from observations and interviews. Data were entered into Excel, and descriptive statistics (frequency and percentage) were used to summarize qualitative variables.

The risk assessment process involved calculating primary and secondary risks, with control measures proposed for risks exceeding acceptable thresholds.

The MIL-STD882E standards guided the classification and prioritization of risks, ensuring a systematic approach to risk management.

## Results

**Figure-1:**
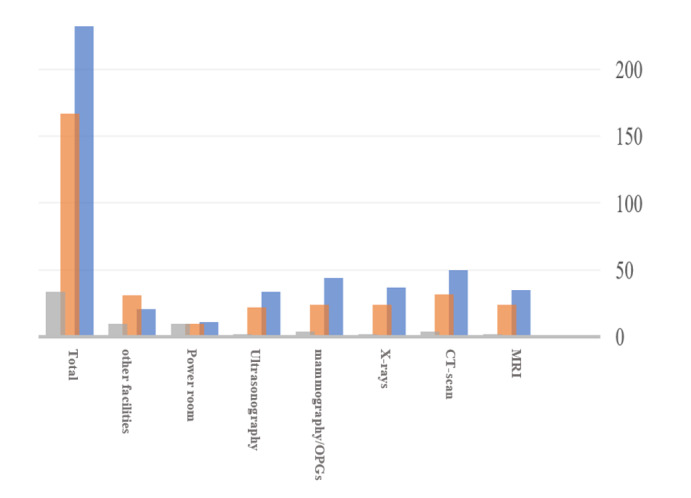
Figure 1. Distribution of risk frequency in different units

**Figure-2:**
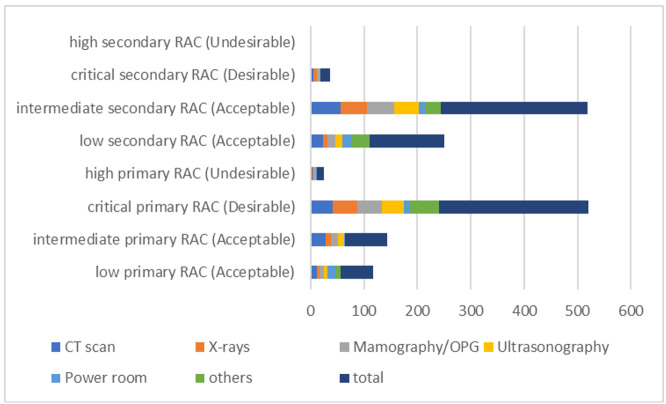
Figure 2. Results of the analysis of primary and secondary risk levels in different units

**Table:** Table1. Overall Results of Risk Assessment in Different Units

Unit	Consequential risks for				Primary risk level								RAC Secondary
	Type of energy	humans	Safety (equipment)	Unacceptable (high) Environment	Total No. Of risks	Unfavorable (serious)	Acceptable (average)	Acceptable (low)	Unacceptable (high)	Unfavorable (serious)	Acceptable (average)	Acceptable (low)	
MRI	10	35	24	2	61	1	41	14	5	0	0	30	31
CT Scan	10	50	32	4	86	4	42	28	12	0	6	56	24
Radiology	9	37	24	2	63	2	46	10	5	0	6	49	8
Mammography and OPG	10	44	24	4	72	4	46	14	8	0	6	52	14
Ultrasound	10	34	22	2	58	0	41	11	6	0	0	45	13
Power room	4	11	10	10	31	2	12	2	15	0	0	12	19
Other	4	21	31	10	62	0	53	0	10	0	0	30	32
Total	57	232	167	34	433	13	281	79	61	0	18	274	141

**Table:** Table2. Frequency of types of energy, their sources, and the number of risks

**Hazard Type**	**Clinical Sources**	**Associated Risks (n)**
**Kinetic Energy**	Moving parts (MRI, CT, Radiology, Ultrasound)	15
**Ionizing Radiation**	X-ray, CT, Mammography, OPG machines	20
**Non-Ionizing Radiation**	Magnetic fields (MRI), UV from monitors, Ultrasound waves	30
**Light Exposure**	Room lighting	10
**Fall Risk**	Patient beds, mobility devices	16
**Cold Exposure**	MRI machine cooling systems	1
**Noise Exposure**	MRI acoustic noise, equipment fans	2
**Biological Hazards**	Microbial/fungal contamination (all units)	20
**Electrical Hazards**	Medical equipment, wiring, electrical panels	240
**Chemical Hazards**	Disinfectants, contrast agents, cooling gels	26
**Impact Hazards**	Collision with machine components	7
**Pressurized Gas Hazards**	Oxygen/helium cylinders	4
**Thermal Burns**	Ultrasound probes, heating devices	30
**Environmental Hazards**	Slippery floors	15
**Total Hazards**	14 distinct hazard types identified	433 total risks

The imaging department included 5 radiologists, 17 radiographers, 9 receptionists, and 2 typists, providing services across different departments and shifts. The department was equipped with 7 devices: a 16-slice CT scanner, an ultrasound machine, a 256-slice CT scanner, an MRI machine, a digital ceiling-mounted radiography unit, an OPG (dental radiography) machine, and a mammography machine.

### MRI Unit Risk Analysis

The MRI unit contained multiple energy-related hazards, including kinetic energy, ionizing/non-ionizing radiation, falls, cold, noise, biological materials, electricity, and chemicals. A total of 35 human-related risks, 24 equipment-related risks, and 2 environmental risks were identified. Risk levels were categorized as: 1 unacceptable, 41 undesirable, 14 acceptable with revision, and 5 acceptable. Most risks were mitigated to acceptable levels with existing controls.

### Digital Radiography Unit Risk Assessment

This unit involved 9 energy types, including kinetic energy, ionizing/non-ionizing radiation, falls, lighting system glare, electricity, disinfectant chemicals, microorganisms, and miscellaneous energies. 63 total risks were identified (37 human, 24 equipment, 2 environmental), with risk levels as follows: 2 unacceptable, 46 undesirable, 10 acceptable with revision, and 5 acceptable. Post-control measures, most risks were reduced to acceptable levels.

### CT Scan Unit Risk Assessment

The CT unit (16-slice and 256-slice machines) had 10 energy types, including kinetic energy, ionizing/non-ionizing radiation, light, electrical components, microorganisms, disinfectants, contrast agents, falls, and miscellaneous energies. 37 human-related, 24 equipment-related, and 2 environmental risks were found. Risk levels included 4 unacceptable, 42 undesirable, 28 acceptable with revision, and 12 acceptable.

### Mammography & OPG Unit Risk Assessment

This unit contained 10 energy types, such as kinetic energy, ionizing/non-ionizing radiation, light, falls, biological hazards, electricity, chemicals, potential energy, and miscellaneous energies. 44 human-related, 24 equipment-related, and 4 environmental risks were identified. Risk levels were 4 unacceptable, 46 undesirable, 14 acceptable with revision, and 8 acceptable.

### Ultrasound Unit Risk Assessment

The ultrasound unit had 10 energy types, including kinetic energy, ultrasonic/ultraviolet waves, light, electricity, heat, biological materials, potential energy, chemicals, and miscellaneous energies. 34 human-related, 22 equipment-related, and 2 environmental risks were noted. Risk levels were 41 undesirable, 11 acceptable with revision, and 6 acceptable, with most mitigated to acceptable levels post-controls.

### Power Room Risk Assessment

This unit involved 4 energy types (chemical, electrical, mechanical, pressurized gas). Across 2 power rooms (6 electrical panels + other sources), 31 total risks were identified (11 human, 10 equipment, 10 environmental). Risk levels included 2 unacceptable, 12 undesirable, 2 acceptable with revision, and 15 acceptable, all mitigated to acceptable levels post-controls.

### Heating/Cooling & Other Sources Risk Assessment

These sources contained 4 energy types (electricity, heat, chemicals, pressurized gas). Across 10 electric heaters, 10 splits, and 20 electrical sources, 21 human-related, 31 equipment-related, and 10 environmental risks were found. Risk levels were 53 undesirable and 10 acceptable, all reduced to acceptable levels post-controls.

Overall findings revealed a total of 433 risks identified (232 human, 167 equipment, 34 environmental), with initial risk levels categorized as 13 unacceptable, 281 undesirable, 79 acceptable with revision, and 61 acceptable; post-control measures, risks were reduced to 18 undesirable, 274 medium (acceptable with revision), and 141 low (acceptable). The CT scan unit had the highest risk burden (86 risks) due to dual machines and operational complexity, while the power room was the lowest-risk unit (31 risks). Clinically, while controls mitigated most risks, some units retained unacceptable risks, warranting further intervention, particularly in the CT scan, mammography/OPG, and radiology units, which require heightened scrutiny due to their elevated risk levels (Table-1). A total of 433 potential hazards or risks were identified in the medical imaging department, with the highest number being 86 for CT scans, followed by 72 for mammography/OPGs, 63 for X-rays, 62 for other facilities, 61 for MRI, 58 for sonography, and the lowest being 31 for power room.

Figure-1 illustrates the frequency of risk types in different units. Accordingly, the most common types of risks had human consequences and equipment impacts, which varied across units. The highest risk with human consequences and safety consequences was observed in the CT scan unit, while the highest risk with environmental impacts was found in the power room.

Figure-2 outlines the results of the analysis of the levels of primary and secondary risks in various units and the total. Accordingly, most of the risks are at an acceptable level after considering the effectiveness of existing controls, so that the primary undesirable risks have decreased from 281 (excluding controls) to 18 undesirable ones, and the other risks are at acceptable levels. For primary risks, the majority fall within the critical RAC (281 instances, deemed Desirable), followed by intermediate (79, Acceptable) and low (61, Acceptable), with minimal high RAC occurrences (13, Undesirable). Notably, the CT scan, X-rays, and Mammography/OPG units exhibit the highest critical primary risks (42, 46, and 46, respectively), while Ultrasonography and "others" show no high primary risks. Secondary risks predominantly align with intermediate RAC (274, Acceptable) and low RAC (141, Acceptable), with a smaller critical subset (18, Desirable) and no high RAC cases. The Power Room and "others" units display lower secondary critical risks (0 and 0), contrasting with imaging units like CT scans (6) and X-rays (6). Table-2 reports the types of energy and their sources as well as the number of risks resulting from each in the units studied. Accordingly, a total of 14 types of energy and 433 risks were identified, with electricity claiming the largest share among the types of energy with 240 cases. This covers electrical equipment, switches and sockets, cables, and electrical panels, which play a key role in all units. Furthermore, some types of energy include those related to biological, chemical, etc.

## Discussion

In this study, a total of 433 risks related to 14 types of energy were identified in different imaging units. The highest risks were linked to electrical energy (240 items), covering electrical equipment, switches, sockets, electrical panels, and cables. Thereafter, thermal energies and non-ionizing radiation stood in second place with 30 items. These findings highlight the critical need for targeted interventions to mitigate electrical hazards in imaging units, as they represent the most prevalent risk category. Some similar studies have been performed in the literature; the results have shown that electricity is one of the most dangerous and significant risks in the system according to the ETBA technique and has caused serious accidents [13]. A study on examining the status of hospitals at Kerman University of Medical Sciences revealed that none of the hospitals had a favorable safety status. Of the total 72 departments and units in the hospitals studied, slightly more than a quarter (27.8%) had a favorable safety status, with the highest score associated with intensive care units (ICU) (88±7.5) and the lowest score linked to radiology departments (46.1±18.3) [5]. This disparity underscores the urgent need for safety improvements, particularly in radiology departments, which consistently show lower safety scores across multiple studies.

In a study of radiology departments in Isfahan, the overall safety status was 58%, reported as moderate and poor. Of the five safety areas, the highest percentage pertained to physical space and equipment safety, and the lowest percentage was associated with the use of personal protective equipment [14]. Another study in Kurdistan also reported that in 20% of cases, the safety status of radiology departments was poor and in 60% it was moderate [15]. A study in Guilan states that the diagnostic departments of the hospitals covered do not have a desirable level of safety. This study of radiology departments at the University of Guilan was evaluated as "moderate" considering the severity and scope of injury incurrence, and the availability of standard criteria. The main safety and security deficiencies in this department were associated with issues such as the lack of emergency exits, alarm systems, personnel training, and fire extinguishing systems [16]. These consistent findings of moderate to poor safety ratings in radiology departments across different regions suggest systemic issues that require standardized safety protocols and enhanced training programs. However, the findings of a survey at Tehran University of Medical Sciences revealed that radiology departments are completely safe in terms of radiation protection and have a satisfactory safety rating in terms of overall safety, by acquiring 80% of the points [17]. This contrast indicates that effective radiation protection measures can be achieved that provids a potential model for improving overall safety in other radiology departments.

A study in Qazvin performed a risk assessment in the imaging units of three public hospitals using the ETBA method in 2019, identifying a total of 24 risks, of which 7 were unacceptable, 5 were undesirable, and 12 were acceptable with revision. Electromagnetic, electrical, and kinetic energies were the highest levels of hazardous energies, respectively [18]. These results reinforce the prominence of electrical risks in imaging units and suggest that the ETBA method is effective in identifying specific energy-related hazards for targeted mitigation. In Tehran, a study dealt with risk assessment using the ETBA method in the intensive care unit (ICU) of Loghman Hospital. A total of 10 types of energy and 35 hazards were identified, of which 13 hazards were estimated to have an unacceptable risk level, 17 were estimated to be undesirable, and 5 were estimated to be acceptable with revision based on the MIL-STD882B standard table. The most significant and dangerous energies detected in the ICU included the risk of explosion of pressurized cylinders, the risk of electrocution and fire caused by electricity, slipping, patient falling due to lack of bed restraint, infectious agents, and hazards from medical waste [13]. The identification of diverse hazards in ICUs highlights the complexity of ensuring safety in high-stakes medical environments, necessitating comprehensive risk management strategies.

In a study on the central heating system of Shahid Beheshti Hospital in Kashan, a total of 8 energies and 35 potential hazards were identified, of which 12 were estimated to be unacceptable, 20 were estimated to be undesirable, and 3 were estimated to be acceptable. The highest risk levels were associated with chemical energy and electrical energy. Further, 90% of the identified hazards showed unacceptable and undesirable risks [19]. This high proportion of unacceptable risks in hospital infrastructure like heating systems emphasizes the need for regular maintenance and safety audits to prevent catastrophic failures. A study conducted to assess the risk of the electrical system of Najmia Hospital using the ETBA method reported that 97% of the equipment did not have an acceptable risk level. Further, 52% had unacceptable risk, 45% had undesirable risk, and only 3% had acceptable risk [20]. These findings further confirm the pervasive electrical safety challenges across hospital systems, calling for urgent upgrades to electrical infrastructure.

An observational study was performed in a hospital in Eastern India. They employed a risk scoring tool and ranked the hazards based on the risk score. Thirty-eight hazards were identified in the study and categorized into natural, physical, chemical, biological, ergonomic, psychological, and safety categories. Fire and storm hazards showed the highest risk scores [21]. This broader categorization of hazards illustrates the multifaceted nature of hospital safety risks, extending beyond energy-related issues to include environmental and human factors. Since compliance with safety and health principles in hospitals leads to enhanced effectiveness of activities, efficiency, and ultimately productivity, compliance with safety requirements in hospitals requires special attention. This study can assist healthcare managers and officials to design safety improvement programs and focus on the units that have the highest risk. The results of this study can also be a basis for future research in the field of risk assessment in other medical sectors. By integrating findings from these diverse studies, healthcare systems can develop evidence-based safety frameworks to address both common and region-specific risks, ultimately improving patient and staff safety across all hospital units.

Since compliance with safety and health principles in hospitals leads to enhanced effectiveness of activities, efficiency and ultimately productivity, compliance with safety requirements in hospitals requires special attention. This study can assist healthcare managers and officials to design safety improvement programs and focus on the units that have the highest risk. The results of this study can also be a basis for future research in the field of risk assessment in other medical sectors.

Strengths and Weaknesses of the Study

The most important strengths of this study included the use of the ETBA method as a comprehensive and structured method for identifying risks, detailed analysis of energy types and risk levels in the imaging environment, and the provision of practical as well as feasible corrective suggestions for risk mitigation. The most important weaknesses of this study included limited focus on one hospital department (imaging), which may lower generalizability to other departments, lack of assessment of the influence of control measures after implementation, which can be considered in future studies, and the limitation in investigating long-term risks associated with ionizing radiation that have delayed effects.

## Conclusion

Overall, electrical energy and ionizing radiation are the main sources of risk in medical imaging units. Although the implemented controls have been able to improve the level of risks, there is still a need for further optimization in high-risk units such as CT scan and MRI. Implementing the proposed suggestions can have a significant influence on reducing risks and enhancing safety in these environments. Also, a comparative study among devices revealed that devices with a higher level of technology (such as CT scan and MRI) that use ionizing radiation and strong magnetic fields had higher risks than simpler devices such as OPG. This demonstrates the need for greater attention to advanced technologies and proper management of their risks. The most important achievement of this study is providing a comprehensive approach to identifying and managing risks in hospital environments that can be employed as a model for other healthcare centers.

## Conflict of Interest

None.
